# Expression data on liver metabolic pathway genes and proteins

**DOI:** 10.1016/j.dib.2016.01.007

**Published:** 2016-01-13

**Authors:** Mooli Raja Gopal Reddy, Chodisetti Pavan Kumar, Malleswarapu Mahesh, Manchiryala Sravan Kumar, Shanmugam M. Jeyakumar

**Affiliations:** Lipid Biochemistry Division, National Institute of Nutrition, Jamai Osmania, Hyderabad 500007, India

**Keywords:** Vitamin A, Steatosis, PUFA, Lipid, Glycogen, Resolvin

## Abstract

Here, we present the expression data on various metabolic pathways of liver with special emphasize on lipid and carbohydrate metabolism and long chain polyunsaturated fatty acid (PUFA) synthesis, both at gene and protein levels. The data were obtained to understand the effect of vitamin A deficiency on the expression status (both gene and protein levels) of some of the key factors involved in lipogenesis, fatty acid oxidation, triglyceride secretion, long chain PUFA, resolvin D1 synthesis, glucose transport and glycogen synthesis of liver, using modern biology tools, such as quantitative real-time PCR (RT-PCR) and immunoblotting techniques. This data article provides the supporting evidence to the article “Vitamin A deficiency suppresses high fructose-induced triglyceride synthesis and elevates resolvin D1 levels” [1] and therefore, these data may be referred back, for comprehensive understanding and interpretations and for future studies.

**Specifications Table**

**Table:** 

Subject area	*Biology*
More specific subject area	*Nutritional Biochemistry*
Type of data	*Figure*
How data was acquired	*Quantitative real-time PCR (Roche LightCycler 480) and Immunoblotting techniques*
Data format	*Analyzed*
Experimental factors	*Effect of vitamin A-deficient diet on non-alcoholic fatty liver disease (NAFLD)*
Experimental features	*Assessed the expression levels of metabolic pathway genes and proteins of liver*
Data source location	*Hyderabad, India*
Data accessibility	*Data presented in this article*

## Value of the data

• First time data on transcripts and proteins levels, involving multiple metabolic pathways of liver.
• The data is potentially valuable to plan and study the regulation of long chain PUFAs and their active mediator syntheses by other fat-soluble vitamins and micronutrients.
• Data also underscore the importance of, assessing the enzyme activities in metabolic pathways, apart from analyzing their expression levels, at protein or gene or both.
• The data of this article would provide impetus for future studies to understand the diverse interactions between macro-and micro-nutrients, at nutrient-surplus and -deficient conditions in humans.

## 1 Data

Fig. 1 shows the expression levels of various pathway genes of liver. A) *Lipid metabolic pathway*: Sterol regulatory element binding protein 1 (SREBP1), fatty acid synthase (FAS), glycerol 3-phosphate acyltransferase (GPAT), fatty acid binding protein (FABP), diacylglycerol acyltransferase (DGAT), peroxisome proliferator-activated receptor α (PPARα) and microsomal triglyceride transfer protein (MTTP). B) *Lipid droplet-associated genes:* Abhydorlase domain containing protein 5 (ABHD5), perilipin 2, 5 and lipin. C) *Long chain polyunsaturated fatty acid (PUFA) and resolvin D1 (RvD1) syntheses pathway:* Delta-5 desaturase (D5D), delta-6 desaturase (D6D), elongase of very long chain fatty acids 2, 6 (ELOVL 2 & 6), 5-lipoxygenase (5-LPO) and 15-lipoxygenase (15-LPO). D) *Carbohydrate metabolic pathway:* Carbohydrate response element binding protein (ChREBP), sodium-dependant glucose transporter 1 (SGLT1), glucose transporter 8 & 2 (GLUT 8 & 2) and glycogen synthase (GS).

Fig. 2 depicts the liver protein expression data. A) *Lipid metabolic pathway and lipid droplet-associated proteins:* SREBP1, FAS, DGAT1, GPAT1, PPARα, MTTP, ABHD5, perilipin 2, 5 and lipin. B) *Long chain PUFA and RvD1 syntheses pathway:* D5D, D6D, ELOVL 2, 6, 5-LPO and 15-LPO. C) *Carbohydrate metabolic pathway:* GLUT2, GS and phosphorylated GS (pGS).

## 2 Experimental design, materials and methods

### 2.1 Design

Male weanling Wistar rats were fed one of the following diets; control, vitamin A-deficient (VAD), high fructose (HFr) and VAD with HFr (VADHFr) of AIN93G composition, for 16 weeks, except half of the VAD diet-fed rats were shifted to HFr diet (VAD(s)HFr), after 8 weeks period. At the end, animals were sacrificed after over-night fasting, liver was removed and stored at −80 °C until further analysis [1].

### 2.2 Liver gene expression by quantitative real-time PCR (qRT-PCR)

Total RNA from liver was isolated and reverse transcription reaction was performed as described earlier [2]. Quantitative PCR (“real-time”) was performed with LightCycler480 Real Time-PCR system (Roche), using pre-validated probes for rat (UPL probes; Roche) and gene-specific primers. Endogenous expression of acidic ribosomal phosphoprotein (ARPP) was used to normalize the expression data and relative expression level was calculated.

### 2.3 Liver protein expression by immunoblotting

Liver samples (~100–250 mg) were homogenized in T-PER tissue protein extraction reagent (Thermo Scientific, Rockford, USA) supplemented with 5% protease inhibitor and 1% phosphotase inhibitor cocktails. Immunoblotting was performed for various proteins of interest using different fractions as described earlier [2]. β-actin was used as loading control and images were analyzed by using Image J 1.46r software (National Institutes of Health, MD, USA).

### 2.4 Statistical analysis

Statistical significance was determined by one-way ANOVA with *post-hoc* least significant difference (*post-hoc* LSD) test. *P* value at ≤0.05 level was considered significant. IBM SPSS statistics 19.0 software (IBM Corp. Armonk, NY, USA) was used for analyses. Data are expressed as mean±SEM.

## Figures and Tables

**Fig. S1:**
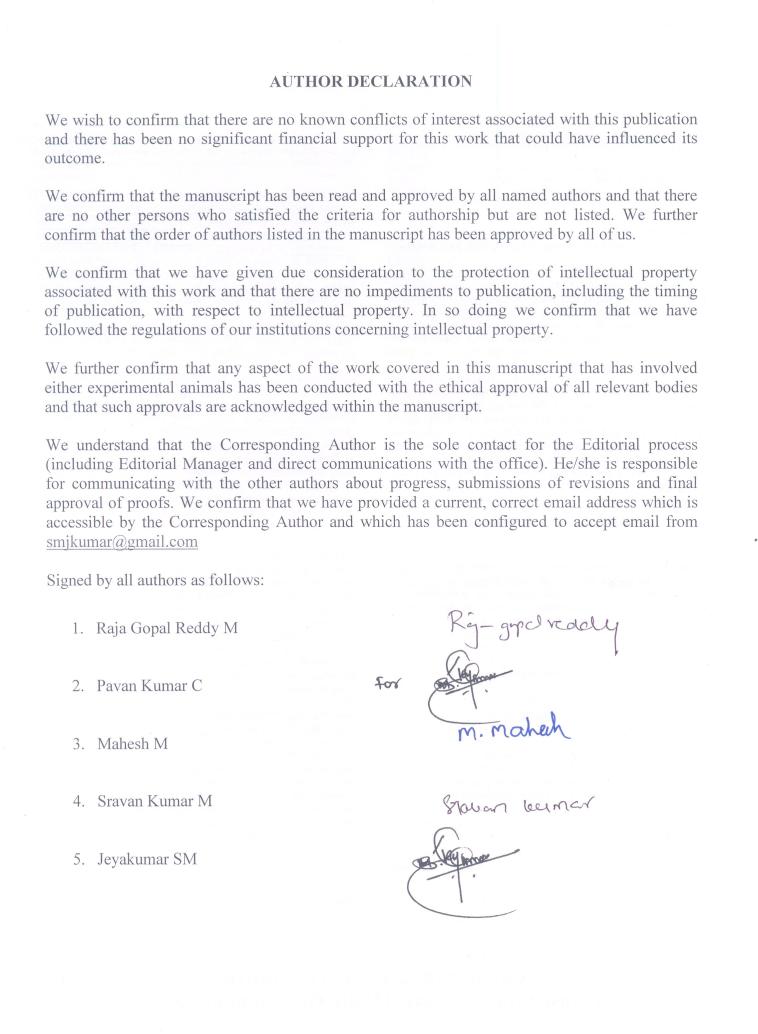


**Fig. 1:**
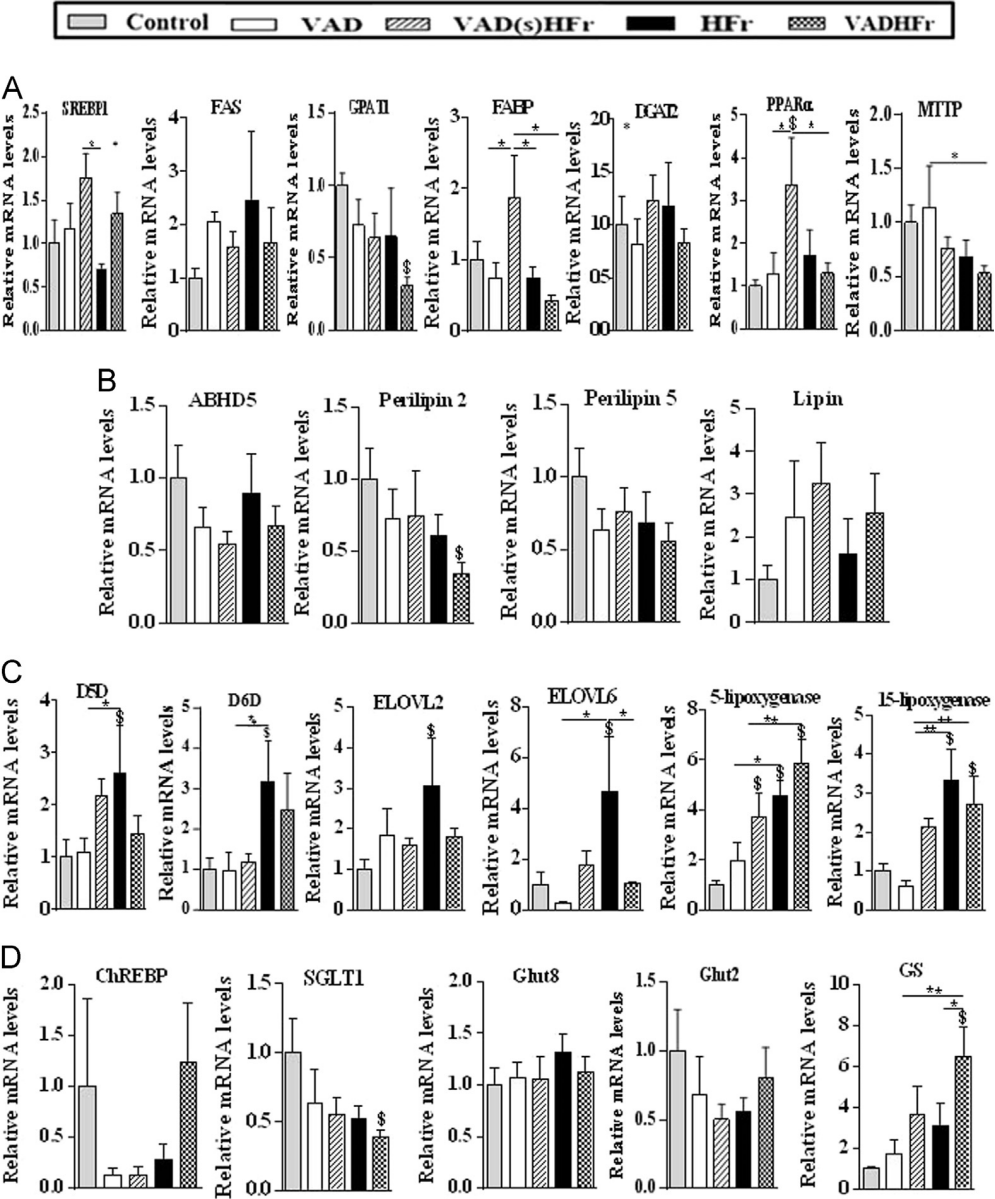
Gene expression of liver metabolic pathways. Relative mRNA levels normalized to control. Data are expressed as mean±SEM (4–5 rats from each group). Data were analyzed by one way ANOVA with *post-hoc* least significant difference (*post-hoc* LSD) test. ^$^ Significant, when compared to control at *P*≤0.05 level. *P* values ≤0.05 and 0.01 levels were denoted as * and ** respectively.

**Fig. 2:**
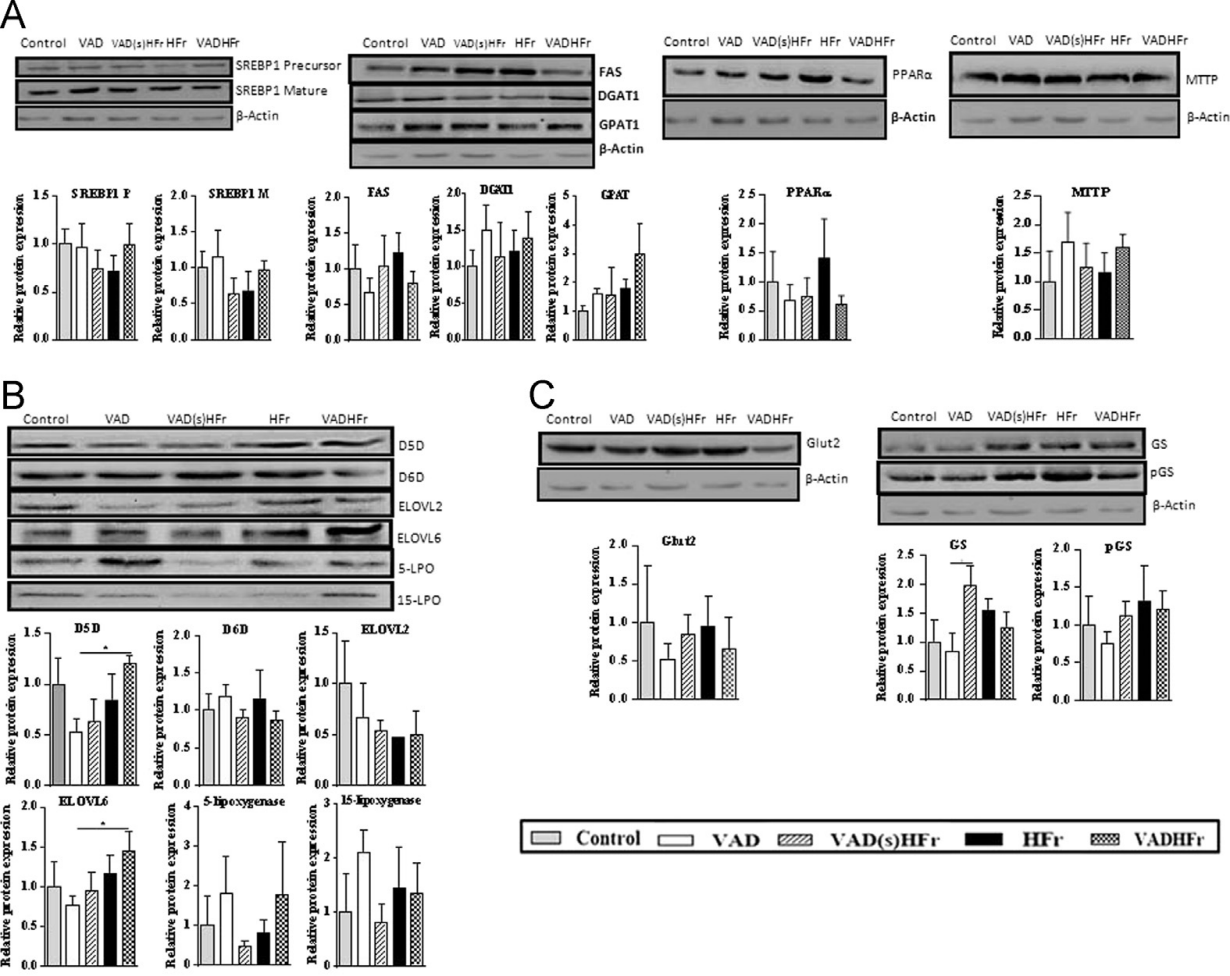
Protein expression of liver metabolic pathways. Representative immunoblots with densitometric analyses data. Data are expressed as mean±SEM (3–4 rats from each group). Data were analyzed by one way ANOVA with *post-hoc* least significant difference (*post-hoc* LSD) test. * Significant at *P* value ≤0.05 level.
